# A synthetic data set to benchmark anti-money laundering methods

**DOI:** 10.1038/s41597-023-02569-2

**Published:** 2023-09-28

**Authors:** Rasmus Ingemann Tuffveson Jensen, Joras Ferwerda, Kristian Sand Jørgensen, Erik Rathje Jensen, Martin Borg, Morten Persson Krogh, Jonas Brunholm Jensen, Alexandros Iosifidis

**Affiliations:** 1 Department of Electrical and Computer Engineering, Aarhus University, Aarhus, 8200 Denmark; 2 Spar Nord Bank, 9100 Aalborg, Denmark; 3 School of Economics, Utrecht University, Utrecht, 3584 EC The Netherlands

**Keywords:** Business, Economics

## Abstract

Bank transactions are highly confidential. As a result, there are no real public data sets that can be used to investigate and compare anti-money laundering (AML) methods in banks. This severely limits research on important AML problems such as efficiency, effectiveness, class imbalance, concept drift, and interpretability. To address the issue, we present SynthAML: a synthetic data set to benchmark statistical and machine learning methods for AML. The data set builds on real data from Spar Nord, a systemically important Danish bank, and contains 20,000 AML alerts and over 16 million transactions. Experimental results indicate that performance on SynthAML can be transferred to the real world. As use cases, we present and discuss open problems in the AML literature.

## Background & Summary

The global framework for anti-money laundering (AML) is regulated by the Financial Action Task Force, requiring that banks monitor and report suspicious transactions^1^. In practice, monitoring is done with electronic AML systems. These often rely on simple business rules, raising alerts for investigation by human bank officers who either (i) dismiss or (ii) report the alerts to national authorities. Most authorities offer little guidance on AML systems, leaving banks to develop them on their own. Complicating matters, there exist no real public data sets with AML bank data^2^. This makes it hard to compare systems and assess their effectiveness, efficiency, and robustness. It also severely limits academic research on open AML problems such as class imbalance, concept drift, and interpretability (see our “Usage Notes” section).

The lack of public AML bank data sets is not without reason. Bank transactions are highly confidential, containing information about sexuality and religious and political affiliations. For financial institutions to publish real data, they would need absolute anonymization guarantees. Unfortunately, the broader scientific literature contains multiple examples of successful de-anonymization attacks^3–7^. In light of this, we argue that simulated or synthetic data is the best viable option for open AML research. Previous work by Lopez-Rojas *et al*.^8^ proposed PaySim, a multi-agent simulator designed to emulate mobile phone transfers. Weber *et al*.^9^ further proposed AMLSim, augmenting and tailoring PaySim to a more classic bank setting where researchers, in addition to simulated normal transactions, can inject (hypothesized) money laundering patterns.

This paper presents SynthAML^10^, a synthetic data set to benchmark statistical and machine learning methods for AML. Our synthetization approach employs the Synthetic Data Vault^11^ (SDV) to tune a probabilistic model with real data. The real data comes from Spar Nord, a systemically important Danish bank with approximately 440,000 clients. SynthAML^10^ contains 20,000 AML alerts and over 16 million transactions in two tables. Tables 1, 2 illustrate the structure of our synthetic (and real) data. The first table holds information about individual AML alerts, including:

1. an alert ID,
2. the date the alert was raised,
3. the outcome of the alert (i.e., if the alert was reported to the authorities or dismissed).

**Table 1:** Alert table (example).

AlertID	Date	Outcome
**1**	2020-01-01	Dismiss
**2**	2020-01-01	Report
**3**	2020-01-02	Dismiss
**4**	2020-01-04	Dismiss
⁝	⁝	⁝

**Table 2:** Transaction table (example).

AlertID	Timestamp	Entry	Type	Size
1	2019-12-28 12:17:13	Credit	Cash	5.70
1	2019-12-28 12:10:49	Credit	Card	2.66
1	2019-12-27 19:33:59	Debit	Wire	1.83
1	2019-12-23 18:01:02	Debit	Wire	1.11
⁝	⁝	⁝	⁝	⁝

The second table holds transaction histories. We have a one-to-many relation where each alert is associated with a sequence of transactions (identifiable though the alert ID number). Each transaction has four features:

1. a transaction timestamp,
2. the transaction entry (credit vs. debit),
3. the transaction type (card, cash, international, or wire), and
4. the transaction size (measured in log Danske Kroner (DKK) and standardized to have zero mean and unit variance).

In both our real and synthetic data, transaction types are encoded to be “mutually exclusive and collectively exhaustive”. We consider any transaction that is not a card, cash, or international transfer to be a wire transfer. This means that transactions made with the popular Danish smartphone app MobilePay (equivalent to the American Venmo or Dutch Tikkie) are encoded as wire transfers. The same holds for checks (although they virtually never are used in Denmark). We define a credit transaction as any transaction that decreases a client’s bank balance. The opposite holds for a debit transaction. We finally stress that definitions of card, cash, and international transfers may vary between banks and even over time within a single bank. For instance, banks may treat transfers to self-governing territories differently and employ different logic regarding canceled or recalled transactions.

## Methods

SynthAML^10^ builds on the SDV library^11^ with conditional parameter aggregation and Gaussian copulas. In the following subsections, we describe (i) our real data, (ii) our synthetization approach, and (iii) our pre- and post-processing steps. The real data was obtained directly from Spar Nord’s internal database. Data access (and usage permission) was obtained as part of some of the authors’ employment at the bank. Because of its sensitive nature, the bank will generally turn down requests to access the real data. Due to confidentiality (and by agreement with the head of the bank’s AML department), we only share our synthetic data; not any real data or code used to transform it. Indeed, providing the real data or our specific transformation implementations would reveal sensitive information about the bank’s internal setup. We do, however, describe our transformations in detail below.

### Real data from Spar Nord

**Fig. 1:**
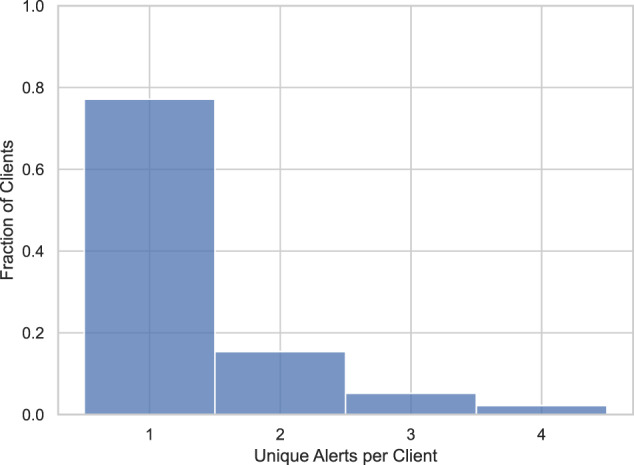
Real alerts per unique client. A little more than 300 clients were associated with more than 4 alerts during the data collection period.

Our real data consists of 20,000 AML alerts sampled from a subset of the rules and models employed by Spar Nord’s AML department. All alerts pertain to private (i.e., non-business) clients and were raised between January 1, 2020, and December 31, 2021. For every alert, we collect all transactions made by the underlying client up to 365 days before the alert was raised. Note that some clients were subject to multiple alerts in the data collection period, see Fig. 1.

For confidentiality, we stratify the real data before we apply our synthetization approach (we always use the label “real data” to refer to the real, non-stratified data). We specifically use bootstrapping (i.e., random sampling with replacement) to ensure that the stratified data contains (i) a 50%–50% split of male and female clients and (ii) a 10%–90% split of high risk vs. non-high risk clients (from an AML perspective). We stress that the chosen proportions not necessarily reflect true client proportions. Furthermore, we emphasize that being a “high risk client” can mean vastly different things in different countries and in different banks. For more information about AML operations and risk ratings in Denmark, we refer to the Danish National Risk Assessment on Money Laundering^12^ and the Financial Action Task Force’s report on AML and counter-terrorist financing in Denmark^13^.

### Synthetization approach

Below, we provide a brief description of our employed synthetization approach with conditional parameter aggregation and Gaussian copulas. For more information, we refer to the original SDV paper^11^.

#### Conditional parameter aggregation

Recall that our real data consists of two tables: a primary table with alerts and a secondary table with transactions (see Tables 1, 2). To capture dependencies between these, the SDV library employs conditional parameter aggregation. The approach iterates over every alert with the following steps:

1. Find all transactions associated with an alert through a conditional lookup on the alert ID in the second table.
2. Perform the Gaussian copula process (see the following subsection) on the resulting transactions, yielding a set of conditional distribution parameters and a conditional covariance matrix.
3. Extend the alert table to hold all conditional parameters found in step 2. Furthermore, we also record the number of transactions associated with each alert.

The extended alert table is then subjected to the Gaussian copula process (see the following subsection). This gives a probabilistic model that accounts for covariances between (i) the original alert features and (ii) the conditional distribution parameters of associated transactions. Simulating an observation is then a two-step process. We first sample an observation from the extended alert table. This immediately yields an alert date and outcome. It also yields conditional parameters that, secondly, are used to simulate associated transactions.

#### The gaussian copula process

Consider a table with *i* = 1, …,*n* observations (i.e., rows) and *j* = 1, …, *m* numerical features (i.e., columns). A generative model for the table may be characterized by:

1. the probability distribution over each feature *x*_*j*_, and
2. the covariance between features *x*_*j*_ and *x*_*h*_ for *j*, *h* = 1, …, *m*, with *j* ≠ *h*.

To capture the distribution of each feature, the SDV library uses the Kolmogorov-Smirnov test^3^ to find the best fit from a set of standard distributions. To capture covariances, the SDV library relies on Gaussian copulas. Let *F*_*j*_ denote the cumulative distribution function (cdf) of feature *j*. It follows from the probability integral transform that *F*_*j*_ (*x*_*j*_) follows a standard uniform distribution. Furthermore, if we let Φ denote the standard Gaussian cdf, we have that $X=\left[{\Phi }^{-1}\left({F}_{1}\left({x}_{1}\right)\right),\ldots ,{\Phi }^{-1}\left({F}_{m}\left({x}_{m}\right)\right)\right]$ follows a multi-dimensional Gaussian distribution. This gives rise to a Gaussian space in which the SDV library estimates a covariance matrix Σ. To synthesize a new observation $x\in {{\mathbb{R}}}^{m}$ (relating to the single table considered), we (i) sample $v \sim {N}_{m}({\bf{0}},{{\bf{I}}}_{m\times m})$, (ii) let *u* = *Lv* where *L* is the Cholesky decomposition such that $L{L}^{{\rm{T}}}=\Sigma$, and (iii) let $x=\left[{F}_{1}^{-1}\left(\Phi \left({u}_{1}\right)\right),\ldots ,{F}_{m}^{-1}\left(\Phi \left({u}_{m}\right)\right)\right]$.

#### Implementation: Pre- and postprocessing

The Gaussian copula process only works with numerical data: a problem when seeking to model datetime features (e.g., alert dates and transaction timestamps) and categorical features (e.g., alert outcomes, transaction types, and transaction entries). To address this and improve the quality of our simulated data, we use a number of feature transformations:

1. To model alert dates, we count the number of days between the date a given alert is raised and January 1, 2020 (making alert dates a numerical feature).
2. To model transaction timestamps, we count the number of seconds between each transaction’s timestamp and the date that any associated alert is raised (making transaction timestamps a numerical feature).
3. To combat skewness and the stylized fact that financial data may span several orders of magnitude, we log transform transaction sizes. Let *t*>0 denote some (absolute) transaction size. We then employ the transformation
  1 $$z={\rm{ln}}\left(t+\varepsilon \right)$$
  where *ε* > 1 is a random constant (fixed for all transactions) we add to allow a positive transformation of transactions smaller than 1 DKK and to preserve confidentiality.
4. To address categorical features (e.g., alert outcomes and transaction types and entries), the SDV library automatically employs numerical replacement. Let *z* ∈ {1, …, *K*} denote a categorical feature that can take *K* distinct values (ordered by decreasing frequencies *f*_1_, …, *f*_*K*_). Now, divide the interval [0,1] into brackets [*a*_*k*_, *b*_*k*_] based on the cumulative probability for each category *k* = 1, …, *K*. For every observation *z* = *k*, the SDV library automatically samples $\widetilde{z}$ from the truncated Gaussian distribution with a mean *μ* and *σ* given by $\mu =\frac{{b}_{k}-{a}_{k}}{2}\,{\rm{and}}\,\sigma =\frac{{b}_{k}-{a}_{k}}{6}$.

When we simulate our synthetic data, we use rejection sampling to ensure that any synthetic alert is associated with a transaction within 7 days (604,800 seconds) of said alert being raised; discarding any synthetic alert and its associated transactions for which this is not the case, instead simulating a new alert and associated transactions.

After running our simulation, we employ a number of postprocessing steps:

1. To convert categorical features back to categorical form, the relevant brackets found during numerical replacement are used (this is done automatically by the SDV library).
2. To convert alert dates back to datetime format, we consider January 1, 2020, and count forward the number of simulated days for each alert. For confidentiality, we also add some random noise. Specifically, we replace the date that any synthetic alert is “raised” with a random date from the same quarter (all dates in the quarter having equal probability).
3. To convert transaction timestamps back to datetime format, we consider the date that any associated alert is raised and count back the number of simulated seconds. For confidentiality, we also add some random noise to all transaction times.
4. To improve simulation quality, we correct the means and variances of the synthetic transactions to approximately match the real transactions per transaction type, entry, and associated alert outcome. This is done under noise in the synthetic alert outcomes. Let *r* ∈ {0,1} denote that an alert is reported (with *r* = 1), we then add noise by updating
  2 $$r=r\left(1-b\right)+\left(1-r\right)b$$
  where *b* follows a Bernoulli *B*(*p*) distribution (*p* is undisclosed for confidentiality). After adding this noise, we, for example, consider all debit wire transactions associated with reported alerts (i.e., where *r* = 1). Let ${s}_{R}^{2}$ and ${s}_{S}^{2}$ denote the variances of the real and synthetic such transactions. We then correct the synthetic transactions *z* according to
  3 $$z=z\times \sqrt{\frac{{s}_{R}^{2}}{{s}_{S}^{2}}}.$$
  Next, we compute the means *m*_*R*_ and *m*_*S*_ of the real and synthetic transactions in question and update the synthetic transactions *z* according to
  4 $$z=z+\left({m}_{R}-{m}_{S}\right).$$
  We stress that the noisy synthetic alert outcomes obtained from (2) only are used to correct means and variances; they are not reflected in the synthetic alert outcomes in SynthAML^10^.
5. For confidentiality, we add some random noise to all transaction sizes. Specifically, each transaction is multiplied by a random number drawn from a U(0.98,1.02) distribution.
6. We clip the synthetic transactions such that the maximum of these, per type and entry, roughly match the real transactions. We also apply a lower clipping (uniform to all synthetic transactions) to keep the minimum transaction size (corresponding to 0.01 DKK) confidential.
7. Finally, we standardize all transactions to have a mean of zero and unit variance.

## Data Records

SynthAML is stored at figshare^10^. The data consists of two files: “synthetic_alerts.csv” and “synthetic_transactions.csv” corresponding to Tables 1, 2. The former file contains information about individual AML alerts, including, for each alert, an ID, a date when the alert was raised, and an outcome of the alert (i.e., if the alert was reported to the authorities or dismissed). The second file contains transaction histories with, for each transaction, a timestamp, an entry type (credit vs. debit), a transaction type (card, cash, international, or wire), and a transaction size (measured in log Danske Kroner (DKK) and standardized to have zero mean and unit variance).

## Technical Validation

We validate our synthetic data in two ways. In the first subsection below, we compare the distribution of the synthetic data to the real data. In the second subsection, we conduct a series of machine learning experiments to investigate whether performance on the synthetic data can be transferred to the real world.

### Distributional comparison with the real data

**Fig. 2:**
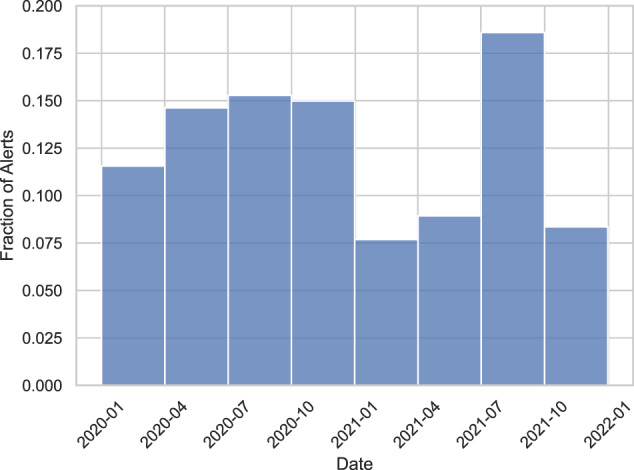
Real alerts raised per quarter throughout 2020 and 2021. Noise is added to keep the exact fractions of alerts per quarter confidential.

**Fig. 3:**
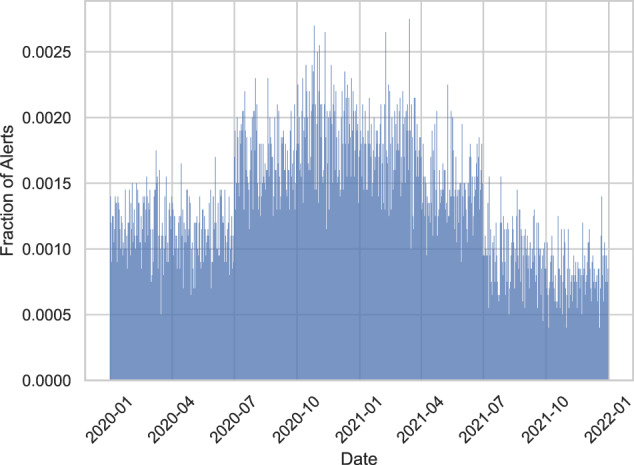
Synthetic alerts raised per day.

**Fig. 4:**
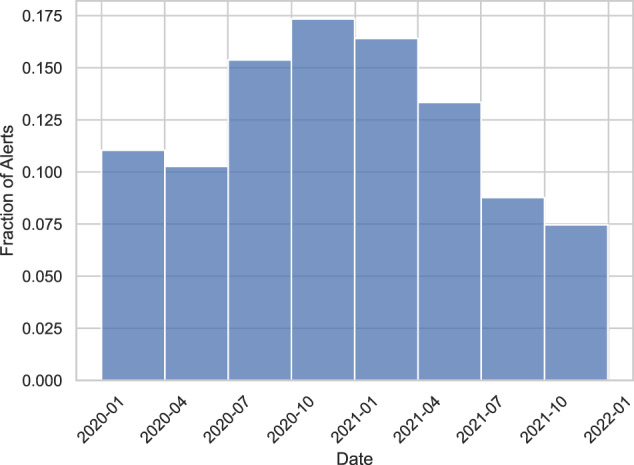
Synthetic alerts raised per quarter.

Figure 3 displays the number of synthetic alerts “raised” per day. Per our synthetization approach, the dates are only informative up to a quarterly division. Thus, Fig. 4 displays the number of synthetic alerts raised per quarter. Compared with Fig. 2, our synthetic dates appear to follow a normal distribution around New Year’s Eve 2021. We believe this is due to our use of Gaussian copulas. However, we do note that AML operations have a seasonal nature: end-of-year financial activity tends to cause many alerts right before and right after New Year’s Eve.

**Fig. 5:**
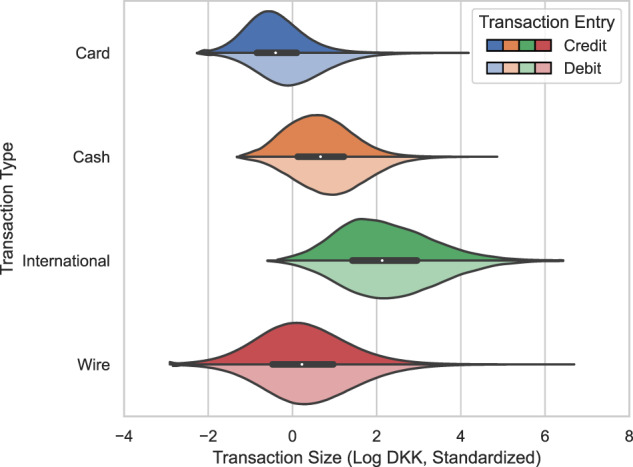
Synthetic transaction size per transaction type and entry.

**Fig. 6:**
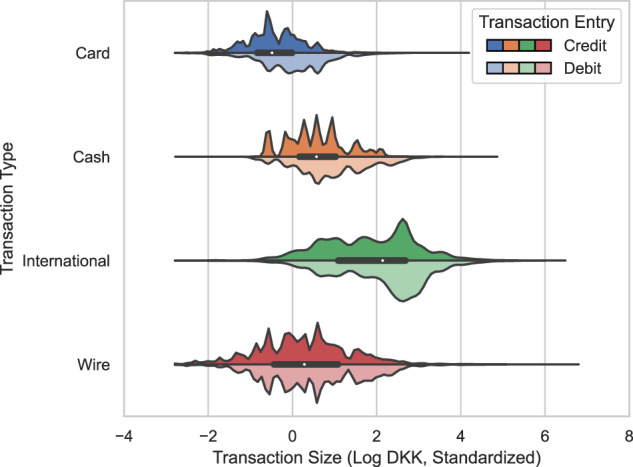
Real transaction size per transaction type and entry. Noise is added to keep minimum and spike values confidential in transformed space.

Figures 5, 6 illustrate the distribution of transaction sizes in our synthetic and real data per transaction type and entry. Notably, the real transactions appear to follow spiked distributions. We believe this reflects that bank clients have a tendency to make round, integer transactions (say, cash withdrawals of 100, 200, 500, or 1,000 DKK). In our synthetic data, however, the distributional spikes and asymmetrical relations between credit and debit transactions are largely removed. Also, note that the synthetic card, cash, and international transactions lack their left distribution tails.

**Fig. 7:**
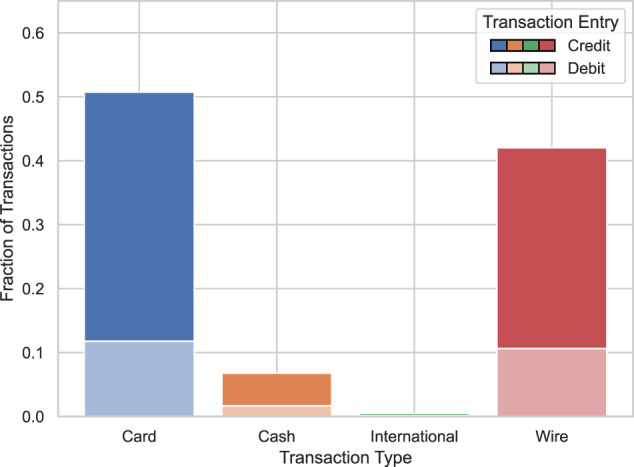
Synthetic transactions per type and entry.

**Fig. 8:**
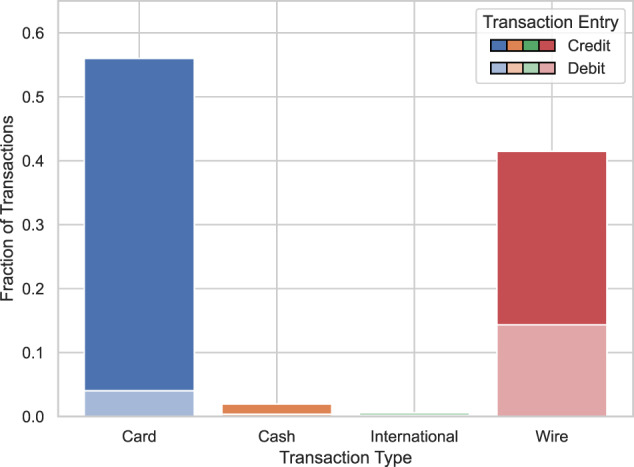
Real transactions per type and entry. Noise is added to keep exact type and entry fractions confidential.

Figures 7, 8 display the distribution of the transaction types and entries in our synthetic and real data. The cash transactions appear overrepresented in our synthetic data. Furthermore, the card debit transactions appear overrepresented while the wire debit transactions appear undersampled.

### Machine learning experiments: Performance transferability

To investigate if performance on SynthAML can be transferred to the real world, we conduct machine learning experiments. The motivating idea is straightforward: train models on the synthetic data and see how they perform on the real data. To provide a baseline, we also train and test models exclusively on the real data.

All our models seek to classify alerts based on their outcomes. We use the same train-test split on both the synthetic and real alerts. As training data, we use alerts raised between January 1, 2020, and December 31, 2020. As test data, we use alerts raised between January 1, 2021, and December 31, 2021. Note that we use all alerts to simulate our synthetic data. Strictly speaking, this introduces a form of target leakage. However, we are not principally interested in predicting alert outcomes. Rather, our machine learning experiments aim to justify that performance on the synthetic data is transferable to the real world.

As features, we calculate the (i) minimum, (ii) mean, (iii) median, (iv) maximum, (v) standard deviation, (vi) count of, and (vii) sum per transaction type and entry for all transactions associated with each alert. This gives 7 × 2 × 4 = 56 features per alert. If a given alert is not associated with any transactions of a particular type and entry, we set the count of such transactions equal to zero. All other features associated with the transaction type and entry (e.g., the average transaction size and median) are set equal to −3 (note that the minimum transaction size of any transaction in our synthetic data approximately equals −2.9). Finally, we scale all features to be mean zero and have unit variance using the training data.

Our experimental protocol makes no attempt to tune model hyperparameters. Unless explicitly stated below, we always keep all hyperparameters at the default values provided by the implementing libraries (library versions are listed in our “Code Availability” section). We consider the following six models:

1. a simple decision tree,
2. a random forest,
3. a logistic regression,
4. a support vector machine,
5. a multilayer perceptron with two hidden layers of 32 neurons using ReLU activation functions,
6. gradient boosted trees implemented with LightGBM.

The first five models are all implemented with the Scikit-learn library, the latter with the LightGBM library (see our “Code Availability” section for versions and links). For the logistic regression and multilayer perceptron, we allow a maximum number of 10^6^ iterations to ensure convergence. All models are fitted and tested using ten different seed values, permutating the training data before each run. Motivated by the class imbalance in our synthetic data (containing approximately 17% reported alerts; not necessarily reflecting the proportion of reports in our real or stratified data) we use the area under receiver operating characteristic curves (ROC AUC) as an evaluation metric^14^. A receiver operating characteristic curve plots true positive versus false positive rates at various classification thresholds for a given model. The area under the curve (often just denoted as AUC) is then a one-dimensional measure of separability; a score of 50% implies a random classifier while a score of 100% implies a perfect classifier.

Table 3 displays our results. Importantly, the relative ranking of the models trained on the synthetic data appears to be consistent: the better a given model performs on the synthetic test data, the better it also performs on the real test data. The relationship does not hold exactly when we consider models trained and tested on the real data. Furthermore, the decision tree, multi-layer perceptron, and random forest are associated with relatively large standard deviations. Finally, we note that all models trained on the synthetic data generally perform worse than models trained on the real data. Still, the results indicate that performances on SynthAML can be transferred to the real world.

**Table 3:** Mean ROC AUC scores (standard deviations in parenthesis), ordered by synthetic test performance.

Data	Model	Synthetic Test Data	Real Test Data
Synthetic Training Data	Decision Tree	52.00 (00.27)	52.26 (00.84)
	Support Vector Machine	56.30 (00.01)	54.85 (00.01)
	Multi-layer Perceptron	57.27 (00.82)	58.40 (03.57)
	Random Forest	62.62 (00.34)	58.87 (00.71)
	LGBM	63.69 (00.00)	63.09 (00.00)
	Logistic Regression	64.10 (00.00)	64.48 (00.00)
Real Training Data	Decision Tree	—	56.35 (00.29)
	Support Vector Machine	—	67.55 (00.01)
	Multi-layer Perceptron	—	66.50 (00.74)
	Random Forest	—	74.99 (00.36)
	LGBM	—	75.55 (00.00)
	Logistic Regression	—	74.75 (00.00)

Alerts raised between January 1, 2020, and December 31, 2020, are used for training. Alerts raised between January 1, 2021, and December 31, 2021, are used for testing.

## Usage Notes

Our results, indicating that performance on SynthAML can be transferred to the real world, imply that SynthAML may be used to investigate a number of open problems in the AML literature. Here, we specifically focus on class imbalance, concept drift, and interpretability. Regardless of the addressed problem, we stress that the synthetic alert dates only are accurate up to a quarterly division: any train-test split of the data should respect this (i.e., splits should be made either January the 1st, April the 1st, July the 1st, or October the 1st). We also stress SynthAML is based on investigated AML alerts. Thus, clients that have never been subjected to alerts are not represented in the data. This is a potential selection bias, although we argue that the approach is reasonable; the alternative is a set of non-investigated clients without labels.

Class imbalance refers to the empirical fact that benevolent bank clients far outnumber money launders. While good for society, this is a potential problem when we train models to flag money laundering behavior. SynthAML contains approximately 17% reported alerts. This is considerably more than in real AML settings (a result of our stratification and synthetization approach), where false positive rates can be 95% to 98%^15^. To investigate the impact of class imbalance, one may subset multiple different training data sets with different proportions of reported alerts. Possible mitigation strategies include under-, over-, and synthetic minority oversampling^16,17^.

Concept drift refers to the empirical fact that transaction and money laundering behavior changes over time. To investigate this, one may, for example, use alerts from one quarter to predict alert outcomes in multiple future quarters. A significant decrease in the test performance between the first and last test quarter would indicate the presence of concept drift. Possible mitigation strategies include active learning^18^ and periodic retaining.

Interpretability is a contested concept within machine learning with multiple overlapping (and sometimes vague) definitions^19,20^. Loosely speaking, the term may be understood as “the degree to which a human can understand why a particular prediction or decision is produced by a model”. In an AML context, this appears very beneficial. To investigate how advanced machine learning models for AML can be made “interpretable”, a researcher may apply different interpretability techniques like local interpretable model-agnostic explanations^21^, Shapley additive explanations^22^, or layer-wise relevance propagation^23,24^ to models trained on SynthAML^10^.

### Considerations on the adversarial nature of anti-money laundering

Results from our technical validation might prompt a concern: could a synthetic AML data set be employed by money launderers to adjust their modus operandi and avoid detection? To answer this, note that all our data have undergone non-invertible transformations. In addition, our data stems from actual AML alerts raised and inquired at Spar Nord. Thus, it would be a bad idea for any criminals to mimic behavior present in SynthAML^10^. Furthermore, our data pertains to a random subset of alerts raised on a subset of the alert criteria and models employed by Spar Nord (i.e., alerts are also raised on behavior absent in SynthAML^10^). Thus, a money launderer cannot ensure that he or she evades detection by displaying a behavior absent in SynthAML^10^. On a more principal note, citing Claude Shannon (on cryptography), we believe that “one ought to design systems under the assumption that the enemy will immediately gain full familiarity with them”. Certainly, examples of insiders helping criminals with information about AML and financial systems are plentiful^25–27^. In light of this, the lack of public AML data only seems to hinder the development of good AML systems and aid money launderers.

## Data Availability

All our simulations are made using version 0.14.1 of the SDV library (https://sdv.dev). We specifically employ the HMA1 model class using two tables as inputs: a primary table with alerts (see Table 1) and a secondary table with transactions (see Table 2). A demonstration by the SDV developers is available online (https://sdv.dev/SDV/user_guides/relational/hma1; using data different from ours). Due to confidentiality, we do not share our code that (i) transforms the raw data so that it can be fed to the HMA1 model class and (ii) re-transforms and adds noise to the simulated data. The data-providing bank felt that providing this code would reveal sensitive information about its internal setup and the real data. All our transformations are, however, described in detail in our subsection “Implementation: Pre- and Postprocessing.” Our machine learning experiments were conducted with version 1.1.3 of the Scikit-learn library (https://scikit-learn.org) and version 3.3.3 of the LightGBM library (https://lightgbm.readthedocs.io/en/stable).
